# Monetary policy responses to COVID-19 in emerging European economies: measuring the QE announcement effects on foreign exchange markets

**DOI:** 10.1007/s10663-023-09578-9

**Published:** 2023-05-14

**Authors:** Idil Uz Akdogan

**Affiliations:** School of Slavonic and East European Studies, University College London, Gower Street, London, WC1E 6BT UK

**Keywords:** Large-scale asset purchases, Exchange rates, Emerging markets, Event study, Volatility, E52, E58, F31, G14

## Abstract

This paper examines the effects of quantitative easing (QE) announcements by emerging market central banks in Europe during the COVID-19 pandemic, particularly on exchange rates with a higher frequency setting. Two different methodologies are used for analysing the policy announcement effects. The first methodology is the event study method that measures the sample exchange rates’ mean and cumulative mean abnormal return around the time of event. The second one is the time series approach that measures asymmetric behaviour of the exchange rate volatility to monetary policy shocks by employing exponential GARCH model. The results show that the foreign exchange markets respond to QE announcements in all selected countries. The response of exchange rates varies across countries and event windows. QE announcements cause appreciation of domestic currency in Hungary and Poland, and depreciation in Turkey. Additionally, the QE announcements increase exchange rate volatility in Hungary and Poland while they reduce volatility in Turkey. The asymmetric behaviour of domestic currencies prevails in all selected countries, but this asymmetry is sensitive to the exchange rate and the length of the window.

## Introduction

The policy response by central banks (CBs) to the economic and financial fallout of the recent pandemic is multifaceted. According to the IMF (2020), 90% of CBs used conventional methods such as cutting policy rates to mitigate the sudden and unprecedented shock to economic activity. Central banks also employed unconventional methods to manage stability in the financial markets. Amongst these methods, quantitative easing (QE) was the most frequently used one as it aimed to provide liquidity to stressed financial markets and influence interest rates other than the usual short-term official rates. The QE implementations became significant especially after the 2007–2009 global crisis. The zero lower bound on nominal interest rate in most developed countries reduced the effectiveness of conventional methods, which later led CBs to prefer QE instead to maintain stability in the financial markets and to promote growth. Subsequently, this resulted in the massive expansion of CBs’ balance sheets across many developed countries with observable impact on financial markets and more complicated effects on growth and inflation rates (Joyce et al. 2012).

The COVID-19 pandemic forced many CBs in emerging economies to intervene to facilitate the smooth functioning of the financial markets and to stimulate the economy. For the first time on a broad basis, at least 18 of these emerging markets’ CBs adopted unconventional policies using large scale asset purchase programs^1^ (IMF 2020). Most of these programmes focused on local currency sovereign bond purchases in secondary markets. The use of QE as an instrument during the pandemic in emerging markets was different than the purpose set by CBs in advanced economies (Arslan et al. 2020). In advanced economies, CBs were designed to provide credit support for firms, keep bond markets functional, and support monetary accommodation as policy rates had reached their effective lower bound. Emerging economies did not explicitly seek to provide monetary stimulus or credit support, but rather addressed market dislocations arising from investor risk aversion. The QE policies in emerging market economies (EMEs) primarily aimed to (1) compensate for the bond sell-off by foreign investors and provide liquidity to the financial sector; (2) prevent surges in local benchmark bond yields; and (3) communicate that CB were ready to purchase their respective sovereign’s assets and temporarily ease government financing pressure without causing any anticipation of a large fiscal expansion that would undermine investor confidence (Arslan et al. 2020; Hartley and Rebucci 2020; IMF 2020). As almost all programmes took the form of outright purchases of bonds financed with reserves, and they were not sterilised. Due to its income risk and change in expectations during the period of uncertainty, foreign exchange rate was one of the most prominent effects of QE. Volatility of the exchange rate, in this respect, was important for the functioning of the monetary policy transition mechanism. It had an imperative role in securing investor confidence and maintaining stability in the financial markets.

The aim of this paper is to study the impact of policy interventions on exchange rate returns during the COVID-19 pandemic, and to measure the volatility of the exchange rates across emerging economies in Europe. Since QE is a new instrument for emerging markets, it provides important information about how the unconventional monetary policies shape expectations in these countries’ financial markets. The analysis is straightforward when assessing the effects of asset purchases programme on the financial markets, particularly around the time of announcements (Di Casola and Stockhammar 2021). Fama et al. (1969) argue that the efficient market is the one that adjusts rapidly to new information. They also argue that asset prices should react immediately to large scale asset purchases (LSAP) news, not just to expected transactions. The important part of the analysis is to observe whether there is an unusual behaviour in the exchange rate after the CB’s announcement for purchasing long term assets in emerging European economies (EEE).

One of the drawbacks of studying the impact of policy implications is that unprecedented times might be complex and, thus, difficult to detect. Along with demand and supply shocks, there could be many other factors simultaneously affecting the asset markets. The advantages of event studies are that they are useful in avoiding other factors affecting the asset markets. Higher frequencies of the event studies with short horizon around an event make the analysis quite reliable (Kothari and Warner 2006). The impact of QE may occur via signalling channel so that the strongest reaction of financial markets is expected to occur upon announcement of the stock purchases, while the execution of the programme is minor in comparison (Urbschat and Watzka 2020). The analysis uses event studies around the time of CB asset purchase announcements, such that the investors price exchange rates mainly based on the information these programmes provide to markets about the expected future path of short-term interest rates. The CB press releases are used for detecting the impact of policy announcements on the domestic currency against euro and the US dollar at a daily frequency for various event windows. The size used to measure the reaction of exchange rates is crucially important as when it is too short, the full market reaction will be missed, and when it is too long, there will be a risk of other factors driving the observed response (Joyce et al. 2012). Thus, different event windows are used in this study to overcome such constraints. Since efficient markets react to news regarding future asset values, not to expected transactions, the focus of this paper is to measure the impact of policy announcement on exchange rate movements and their volatility. The analysis also covers the impact of QE announcements on the 10-year government bond yields for the robustness of the results.

The impact of QE on domestic and foreign asset markets has been analysed extensively. However, now that QE is a new instrument for CBs in emerging markets, the impact of QE on the financial markets is unknown or very limited. This paper aims to fill this gap by analysing the impact of emerging economies’ QE programmes on exchange rates and volatility. Since the purpose of the QE programmes is different in emerging economies than it is in advanced economies, it is important to analyse how CBs use instruments to maintain stability in the financial markets during a catastrophe. Furthermore, the impact on exchange rates can be useful in analysing the effectiveness of the CB policies through signalling channel. Exchange rates fluctuates larger in emerging economies, and CBs use foreign reserves to manage these fluctuations. Therefore, the response of exchange rates to QE is, a valuable source of information that allows CBs to observe the transmission mechanism and CB communication more deeply. It will be useful to see whether any further action is required by the CBs to even out the unintended consequences of such policies on exchange rates. It also provides information regarding the efficiency of the foreign exchange markets to assess how CBs react to a new information in EEE.

The rest of the paper is organised as follows: Sect. 2 provides literature review; Sect. 3 explains the relationship between the asset purchases and the exchange rates, Sect. 4 presents event study methodology and estimation results, and Sect. 5 presents the exponential GARCH model for measuring event-induced volatility. Finally, Sect. 6 will present the conclusion.

## Literature review

Since the start of the first QE programme in 2001 by the Bank of Japan, there had been growing interest in literature to study the effects of QE programmes in asset markets. However, after the financial crisis of 2008–2009, the Fed, the BoE, the Bank of Japan, and the ECB all started various asset purchasing programmes as an example of unconventional monetary policy instrument. The studies in general examined the impact of QE on bond yields and exchange rates. The QE programmes were expected to reduce bond yields and stimulate growth. There were studies such as Neely (2011) and Gagnon et al. (2011) explaining the effects of Fed’s large-scale asset purchases (LSAP) on asset prices in the US. Gagnon et al. (2011) argued that the Fed’s QE programs during the post crisis period led to economically meaningful and long-lasting reductions in longer-term interest rates on a varying range of securities. Neely (2011) showed that the Fed’s first program of large-scale asset purchases reduced bond yields not only in the US but also in other countries. Urbschat and Watzka (2020) examined the short-term reaction for the post 2014 period of financial markets after the Asset Purchase Programme of ECB.

The impact of QE programmes on bond yields or prices are straightforward, however, the impact on exchange rates is more complicated. Kenourgios et al. (2015) examined the effects of QE announcement of the ECB, the Bank of Japan, and the Bank of England on exchange rate dynamics by using intraday frequency. They concluded that volatility of the euro increased around the QE announcements of the ECB. On the other hand, the BOE’s and the BoJ’s QE announcements caused appreciation of domestic currencies without increasing volatility. Dedola et al. (2018) studied the impact of QE on the US dollar per Euro exchange rate and their findings suggested that QE measures had large and persistent effects on the exchange rate. Neely (2011) examined the effect of the Fed’s large scale asset purchases (LSAP) on international long term bond yields and exchange rates, and he studied whether the observed behaviour was consistent with a simple portfolio balance model and standard exchange rate parity conditions. Fratzscher et al. (2013) argued that the first LSAP led to fund inflows in US and thus appreciation of the USD, while the second LSAP caused the opposite results. They also suggested that there were global spillover effects and externalities from the US monetary policy decisions in advanced and emerging economies.

The number of studies regarding the impact of QE in emerging economies is limited. These empirical studies generally examine the impact of the developed economies’ QE programs on emerging markets. For example, Kawai (2015) examined the impact of unconventional US and Japanese monetary policies on emerging economies. He argued that after the QE programs in the US and Japan, the tendency of large currency depreciations occurred in emerging economies with large current account deficits, public debt, and high inflation. Economies with sound macroeconomic fundamentals were usually less affected. Fratzscher et al. (2013) found that the response of portfolio rebalancing in emerging markets might vary depending on the US QE policy, and countries failed to shield themselves from spillover effects. Chen et al. (2012) studied the international spillover impact of QE. They found that the impact of the US QE on emerging economies was generally stronger than that of the advanced economies. Gagnon et al. (2017) argued that increases in the US bond yields were associated with increases in foreign bond yields and in stock prices, as well as with depreciations of foreign currencies. They further argued that all the effects were smaller on the day of the US unconventional monetary policy announcements. Tran and Pham (2020) examined the spillover effects of the US unconventional monetary policy on Asian emerging markets. They found that unconventional monetary policy shocks from the US were associated with a surge in equity prices, a decline in long term interest rates, and an appreciation of currencies in Asian developing markets. Hartley and Rebucci (2020) studied the impact of QE on long-term government bond yields in advanced and emerging markets. Their finding was that the average impact of emerging market QE announcements was significantly larger than that in advanced economies. Arslan et al. (2020) argued that local currency bond yields fell significantly following QE programme announcements in emerging market economies, with little effect on exchange rates.

Event study is a popular method in measuring the announcement effects due to its precision in identifying the reaction of asset following each event. Kočenda and Moravcová (2018) analysed how the emerging European forex markets reacted to foreign macroeconomic news and the monetary policy settings of the major CBs. They concluded that QE announcements affected the value of the new EU members’ exchange rates. Fratzscher (2008) studied whether communication and actual interventions in foreign exchange markets were successful in moving exchange rate in the short and long-run. He suggested that exchange rate communication, and to some extent actual intervention, might be an effective policy instrument that had a permanent effect on exchange rates. There was also another event study for measuring the announcement of foreign exchange interventions in transition economies and in Turkey (Ègert 2006).

The next group of studies will focus on the impact of the pandemic on various asset markets. Pandey and Kumari (2021) studied the impacts of the 2019-nCoV outbreak on the global stock markets by using an event study method. They concluded that the outbreak significantly impacted the global stock markets with the Asian stock markets being hit the hardest. Akhtaruzzaman et al. (2021) argued that during the outbreak China and Japan appeared to be net transmitters of volatility spill overs, suggesting that financial contagion followed a similar pattern to that of the virus contagion. Heyden and Heyden (2021) showed how the US and European stock market reacted negatively to the announcement of the first death in a given country, and how monetary policy measures had the potential to calm markets. Finally, Pandey and Kumar (2022) examined the impact of Covid-19 outbreak on the Indian tourism sector by using event studies, and Mishra (2020) analysed the disaster risk management in India during the pandemic.

## The asset purchases and the exchange rates

CB asset purchase programmes are a non-traditional approach to boosting economies, mostly when conventional measures fail to provide stability. There is no easy answer to the question of how much and in what direction these purchases affect not only the bond market, but the foreign exchange markets too. CB asset purchase programmes are akin to any other conventional instrument, and they act as an expansionary policy by raising equity prices and lowering government bond yields. They are also effective in changing the cross-border capital flows. By compressing the excess return on domestic bonds, they encourage investors to rebalance their portfolios towards higher foreign yielding assets. QE is likely to depreciate the exchange rate as domestic wealth holders use liquid to buy foreign assets (Palley 2011). Thus, the asset purchases affect the exchange rate in broadly the same way as conventional monetary policy—especially through expectations of interest rate differentials (Cœuré 2017).

The response of CBs to business cycle fluctuations can vary across different emerging economies. Table 1 shows the correlation between policy rates and the real GDP in five EEE for the last two decades. The negative correlation for the period between 2000–2019 shows that the CBs conduct procyclical monetary policies, i.e., having monetary expansion in good times and contraction in bad times. One important reason for this behaviour of CB is the fear of free falling (Calvo and Reinhart 2002). In most emerging markets, bad times coincide with heavy capital outflow and loss of policy credibility, which further triggers substantial currency depreciation. Table 1 also shows the correlation between policy rates and the real GDP during the 2007–2009 financial crisis, however, only Croatia and Romania conducted countercyclical policies to reverse the impact of crisis and promote growth. However, during the post crisis period (2010–2019), Turkey is the only country that conducted countercyclical policies.

**Table 1:** Correlation between the policy rate and the GDP

	Croatia	Hungary	Poland	Romania*	Turkey
2000–2019	− 0.049	− 0.883	− 0.723	− 0.798	− 0.687
2007–2009	0.282	− 0.102	− 0.275	0.302	− 0.615
2010–2019	− 0.531	− 0.792	− 0.714	− 0.646	0.135

*Data covers 2003–2019

*Source* National Central Banks for policy rates and Eurostat for the GDP

CBs in EME use various instruments to cope with economic and financial repercussions of the sudden shocks in the markets. These instruments include a cut in policy rate, intervention in foreign exchange markets, and providing extra liquidity in asset markets. Liquidity expansion can be conducted through extending existing facilities or setting up new ones, and/or through broadening eligible collateral for repo operations (Arslan et al. 2020). Many emerging countries launched local currency bond purchase programmes with the intention of coping with the difficulties associated with the COVID-19 pandemic. CBs conducted expansionary monetary policies to cope with uncertainty and to restore stability. These policies included conventional instrument, such as policy rate changes and unconventional instrument such as LTAP and foreign exchange operations. Table 2 lists the policy announcement days and the instruments used by EEE during the pandemic.^2^

**Table 2:** Policy announcements

Country	No. of events	Date	QE	Policy rate cut	Forex operations
Croatia	1	13.03.2020	Yes	No	Yes
Hungary	1	24.03.2020	Yes	No	No
	2	28.04.2020	Yes	No	No
	3	21.07.2020	Yes	Yes	No
	4	27.04.2021	Yes	No	No
Poland	1	17.03.2020	Yes	Yes	No
	2	08.04.2020	Yes	Yes	No
	3	28.05.2020	Yes	Yes	No
	4	16.06.2020	Yes	No	No
	5	14.07.2020	Yes	No	No
	6	15.09.2020	Yes	No	No
	7	07.10.2020	Yes	No	No
	8	06.11.2020	Yes	No	No
	9	02.12.2020	Yes	No	No
	10	13.01.2021	Yes	No	No
	11	03.02.2021	Yes	No	Yes
	12	03.03.2021	Yes	No	Yes
	13	07.04.2021	Yes	No	Yes
	14	05.05.2021	Yes	No	Yes
Romania	1	20.03.2020	Yes	Yes	No
	2	29.05.2020	Yes	Yes	No
	3	05.08.2020	Yes	Yes	No
Turkey	1	31.03.2020	Yes	No	Yes
	2	17.04.2020	Yes	No	No

*Source* National Central Banks

CBs in EEE responded quickly to mitigate the shock of COVID-19 on financial markets. In particularly, Poland continuously made announcements that the QE programme would remain to maintain stability in the financial markets. Tables 3 and 4 respectively show the impact of the CB announcements on the US dollar exchange rate and euro exchange rate. The results are provided by using the standard ordinary least square (OLS) estimates that have time dummy variables for the announcement dates with 3-day, 7-day and 11-day event windows. The results provide mix evidence of the impact made by these announcements on exchange rates. For example, the announcements caused depreciation of the domestic currency in Croatia. Yet, in Hungary it caused domestic appreciation for a 3-day window and domestic depreciation for 11-day window. In Poland, the first announcement caused depreciation, but the following announcements caused appreciation.^3^ The simple OLS model was statistically significant only for euro within a 3-day window. Even though the results show varying degrees of impact on different exchange rates, standard time series techniques may not be well suited when dealing with the analysis of policy implications vis-à-vis the behaviour of exchange rates. Exchange rates are typically highly volatile on a day-to-day basis, and the intervention tends to come in sporadic clusters. It may seem less surprising that the time series studies tend not to find strong evidence for a systematic link between exchange rate movements and monetary policy interventions (Fatum and Hutchison 2003).

**Table 3:** Cumulative percentage change in dollar exchange rate

Country		Date	11-Day window		7-Day window		3-Day window	
			Impact	SE	Impact	SE	Impact	SE
Croatia	1	13.03.2020	0.003***	0.001	0.003**	0.001	0.004**	0.002
Hungary	1	24.03.2020	0.008***	0.002	0.003	0.002	− 0.006*	0.004
	2	28.04.2020	0.002	0.002	0.001	0.003	0.002	0.004
	3	21.07.2020	− 0.003*	0.002	− 0.005**	0.002	− 0.005	0.004
	4	27.04.2021	− 0.001	0.002	− 0.001	0.002	− 0.004	0.003
Poland	1	17.03.2020	0.011***	0.002	0.017***	0.002	0.019	0.004
	2	08.04.2020	0.001	0.002	− 0.004	0.003	− 0.004	0.003
	3	28.05.2020	− 0.005**	0.002	− 0.009***	0.003	− 0.007**	0.003
	4	16.06.2020	− 0.001	0.002	0.001	0.002	0.001	0.004
	5	14.07.2020	− 0.001	0.002	− 0.002	0.002	− 0.006	0.004
	6	15.09.2020	0.001	0.002	− 0.001	0.002	0.000	0.004
	7	07.10.2020	− 0.003	0.002	− 0.003	0.003	− 0.001	0.003
	8	06.11.2020	− 0.003	0.002	− 0.005**	0.002	− 0.011***	0.004
	9	02.12.2020	− 0.003	0.002	− 0.004	0.003	− 0.007**	0.003
	10	13.01.2021	0.000	0.002	0.002	0.003	0.001	0.003
	11	03.02.2021	− 0.001	0.002	− 0.003	0.002	0.002	0.003
	12	03.03.2021	0.002	0.002	0.003	0.002	0.004	0.003
	13	07.04.2021	− 0.003*	0.002	− 0.004*	0.002	− 0.006*	0.003
	14	05.05.2021	− 0.001	0.002	− 0.001	0.002	0.002	0.003
Romania	1	20.03.2020	0.003**	0.001	0.002	0.002	0.003	0.003
	2	29.05.2020	− 0.004***	0.001	− 0.005***	0.002	− 0.006**	0.002
	3	05.08.2020	− 0.002*	0.001	− 0.002	0.002	− 0.003	0.003
Turkey	1	31.03.2020	0.004	0.004	0.002	0.005	0.009	0.007
	2	17.04.2020	0.003	0.004	0.005	0.005	0.006	0.007

*^,^**^,^***Denote statistically significance at 10%, 5% and 1%, respectively

**Table 4:** Cumulative percentage change in euro exchange rate

Country		Date	11-Day window		7-Day window		3-Day window	
			Impact	SE	Impact	SE	Impact	SE
Croatia	1	13.03.2020	0.001***	0.000	0.001***	0.000	0.001***	0.000
Hungary	1	24.03.2020	0.005***	0.001	0.002	0.002	− 0.005**	0.002
	2	28.04.2020	0.000	0.001	0.000	0.002	0.001	0.002
	3	21.07.2020	− 0.001	0.001	− 0.003*	0.002	− 0.002	0.002
	4	27.04.2021	0.000	0.001	− 0.001	0.002	− 0.002	0.002
Poland	1	17.03.2020	0.006***	0.001	0.007***	0.001	0.010***	0.002
	2	08.04.2020	0.000	0.001	− 0.001	0.002	− 0.001	0.001
	3	28.05.2020	− 0.002*	0.001	− 0.004***	0.001	− 0.002	0.002
	4	16.06.2020	0.001	0.001	0.001	0.001	0.001	0.002
	5	14.07.2020	0.000	0.001	0.000	0.001	0.000	0.002
	6	15.09.2020	0.001	0.001	0.000	0.001	0.000	0.002
	7	07.10.2020	− 0.002**	0.001	− 0.002	0.001	0.000	0.002
	8	06.11.2020	− 0.002	0.001	− 0.003**	0.001	− 0.003	0.002
	9	02.12.2020	− 0.001	0.001	0.000	0.001	− 0.001	0.002
	10	13.01.2021	− 0.001	0.001	0.000	0.001	0.001	0.002
	11	03.02.2021	0.000	0.001	− 0.002*	0.001	− 0.001	0.002
	12	03.03.2021	0.001	0.001	0.001	0.001	0.002	0.002
	13	07.04.2021	− 0.002**	0.001	− 0.002*	0.001	− 0.003*	0.002
	14	05.05.2021	− 0.001	0.001	− 0.001	0.001	0.002	0.002
Romania	1	20.03.2020	0.000	0.000	0.000	0.000	0.000	0.001
	2	29.05.2020	0.000	0.000	0.000	0.000	0.000	0.001
	3	05.08.2020	0.000	0.000	0.000	0.000	0.000	0.001
Turkey	1	31.03.2020	0.004	0.004	0.003	0.005	0.011*	0.007
	2	17.04.2020	0.003	0.004	0.005	0.005	0.0047	0.0069

*^,^**^,^***Denote statistically significance at 10%, 5% and 1%, respectively

The standard time-series techniques are insufficient when it comes to dealing with data on exchange rates and policy changes. The event study, on the other hand, is a useful methodology for measuring the reaction of the financial assets through observation of the abnormal returns (ARs) following a policy announcement. The crucial step in any event study is to choose “the right” events. It is common to look at official announcements and policy instruments made by the CB to identify these events (see Szczerbowicz 2015; Gagnon et al. 2011). CB announcements may contain information regarding the conduct of QE together with other conventional and/or unconventional monetary policy instruments. When the CB in Croatia had its first (and only) QE programme, it also intervened by selling foreign exchange on three occasions between March 9 and 13 to preserve the exchange rate stability. In another example, the CB in Poland reduced the policy rate during the first three QE programs and conducted foreign exchange operations during the last four QE programs. Thus, it is crucial that this study includes the policy announcements listed in Table 2 when there is only QE in the announcement so that there will be no other policy affecting the behaviour of exchange rates. Consequently, Croatia and Romania are discarded from the analysis as CBs implemented other instruments simultaneously with QE. Since the focus is measuring the impact of QE announcement alone, then the analysis will only include events with QE as the sole policy instrument. This reduces the number of events to be included in the analysis, as a result Hungary has three events, Poland has seven events, and finally Turkey has only one event available to be used in the event study analysis.

## Event study: expected return model

An event study is used to analyse the announcement effects for a short horizon around the CB’s QE policy with its impact on foreign exchange markets. An event study approach was initially proposed by Fama et al. (1969), who made the following critical three assumptions: (1) The event was unexpected, (2) There were no confounding actors impacting the asset prices being studied, and (3) The markets were efficient. In this respect, event studies served an important purpose in asset markets as a way of testing market efficiency. Systematically nonzero ARs that persist after a particular type of event were assumed to be inconsistent with market efficiency. Accordingly, event studies which focus on long horizons could provide key evidence on market efficiency (Brown and Warner 1985; Fama 1991).

Event studies with high frequency data try to precisely measure the rapid asset price changes usually seen after macro announcements (Neely 2011). Thus, the causality runs one way from the announcements to the asset returns. One advantage of the event study approach is that it reduces the dimensionality of measuring the effectiveness of events into a single dimension by distinguishing whether the impact is observed or not. This binominal setting helps to avoid the problem of “noise” affecting the precision of time-series, but also ignores the information about the magnitude of exchange rate movements (Fratzscher 2008). To overcome this drawback, GARCH specification is applied to measure volatility in the second part of the analysis.

Neely (2011) argues that it is difficult to separately quantify the effect on expectations of each announcement because one cannot easily measure QE expectations. Some announcements such as the ones in Turkey may be considered sudden and unexpected, whereas in other countries such as Poland and Hungary, there can be a continuation of policy announcements in the consecutive meeting minutes. In such cases, the policy may be partially expected, and the surprise component may be small. Nevertheless, the Covid-19 outbreak has caused unprecedented disruptions to economies and posed unprecedented changes to policy implementations. QE is a completely new monetary policy instrument for these countries. The conduct of a new instrument during the pandemic when there is high volatility together with uncertainty prevailing over an unforeseen period can restore the surprise component for all recurring policy announcements.

The structure of the tests used for the event study is unique as it is tailored for the purpose of this analysis. The timing of the QE announcements and the number of events vary significantly across countries. Thus, each country is assessed separately to capture any country specific behaviour in the foreign exchange markets. Since some countries exercised the policy multiple times, the analysis aims to measure the overall impact of policy affecting the expectations of the currency traders during the pandemic. This helps to evaluate the performance of the CBs and it also provides useful information for measuring the efficiency of the foreign exchange rate markets during the pandemic.

The null hypothesis (H_0_) for the event study analysis is that the QE announcements have no statistically significant impact on asset returns. In other words, H_0_ states that the mean of the ARs within the event window is zero whereas the alternative hypothesis (H_1_) states the opposite. The estimation window starts from 1 January 2018 to 29 January 2020 with around 305 observations, which model parameters are obtained. Event window has around 260 observations starting from 30 January 2020, the day WTO declared the COVID-19 Pandemic, to 31 May 2021.

All event studies start with determining the AR or excess returns. To do that, one first needs to define returns in the foreign exchange market as follows:

1 $${\text{R}}_{{\text{h}}} = {\text{K}}_{{\text{h}}} - {\text{e}}_{{\text{h}}}$$

where R_h_ stands for the daily changes of returns in the foreign exchange market represented in a logarithmic form as ln(P_h_/P_h–1_), K_h_ is the expected or predicted returns that is calculated by the interest parity condition (IPC) that can be seen as the difference between foreign and domestic interest rates. Given this return decomposition, e_h_, is the difference between the observed return and the predicted return.

2 $${\text{AR}}_{{\text{h}}} = {\text{R}}_{{\text{h}}} {-}{\text{K}}_{{\text{h}}} {-}{\text{e}}_{{\text{h}}}$$

Let *AR*_h_ represent the ARs of asset on day *h*. Under a null hypothesis of no abnormal performance, the event date ARs will have an expected value of zero. To be able to test the null hypothesis, each AR is divided by its estimated standard deviation to yield a standardised AR. Thus, the standardised ARs will be calculated as follows:

3 $$SAR_{{\text{h}}} = AR_{{\text{h}}} /S\left( {AR} \right)$$

S(AR) is the standard deviation of AR from the estimation period and calculated as follows:

4 $$\left( {AR} \right) = \sqrt {\frac{1}{m - k}\mathop \sum \limits_{h = - s}^{{ - m - \left( {s - 1} \right)}} AR_{h}^{2} }$$

where *s* is the number of days between the announcement of the pandemic and the event day, *m* denotes the estimation window length that is the number of non-missing (i.e., matched) ARs in the estimation window and *k* is the number of parameters used in the estimation model. Estimation and event windows are represented as follows:

$${\text{Estimation}}\;{\text{window}}\;{\text{is}}\;{\text{h}} = - {\text{s}}, \ldots - {\text{m}} - \left( {{\text{s}} - {1}} \right)$$

$${\text{Event}}\;{\text{window}}\;{\text{is}}\;{\text{h}} = - 5, \ldots 0,{1}, \ldots {5}$$

Alternatively, let the average AR $\overline{AR}_{n}$ over an event period containing n returns and let $PE(\overline{AR}_{n} )$ denote its prediction standard error (Corrado and Truong 2008).

5 $$PE\left( {\overline{AR}_{n} } \right) = \frac{{SE_{m} }}{\sqrt n }\sqrt {1 + \frac{n}{m} + \frac{{n\left( {\overline{AR}_{n} - \overline{AR}_{m} } \right)^{2} }}{{\mathop \sum \nolimits_{h \in m} \left( {AR_{h} - \overline{AR}_{m} } \right)^{2} }}}$$

Equation 5 shows that ${\overline{AR} }_{m}$ is the average AR over the estimation period, ${\overline{AR} }_{n}$ is the average AR over the event period, and AR_h_ is the AR on day h. The number of event period returns are represented by n, and the number of returns over the estimation period is represented as m. ${SE}_{m}$ denotes the standard error from the estimation period regression. The ratio of ${\overline{AR} }_{n}$ over ${PE(\overline{AR} }_{n})$ represents a standardised excess return. The model has a null hypothesis of no abnormal performance, assuming that the returns are identical, independent, and normally distributed. The standardised excess return ${\overline{AR} }_{n}/{PE(\overline{AR} }_{n})$ is distributed as Student-t with m-2 degrees of freedom (Corrado and Truong 2008, p. 500). The standard error for multiday event analysis, represented in Eq. 5, takes the following form for the standard error in a single day analysis.

6 $$PE\left( {AR_{0} } \right) = SE_{m} \sqrt {1 + \frac{1}{m} + \frac{{\left( {AR_{0} - \overline{AR}_{m} } \right)^{2} }}{{\mathop \sum \nolimits_{h \in m} \left( {AR_{h} - \overline{AR}_{m} } \right)^{2} }}}$$

The following sections will employ a set of parametric and non-parametric test statistics commonly used in short-term event studies. The reason for using different tests is to eliminate a bias that might have occurred by relying on the outcome of only one type of test. All these tests are implemented using spreadsheet software, except the IPC regressions that are used to derive ARs.

- **Parametric T-Test**

One of the widely used parametric test statistics is the classic parametric T-test proposed by Patell (1976) and Dodd and Warner (1983), which is commonly referred as the Patell T-test, *T*_*p*_.

7 $$T_{P} = \frac{1}{\sqrt N } \mathop \sum \limits_{t = 1}^{N} \frac{{\sqrt {n_{t} } \overline{AR}_{nt} }}{{PE\left( {\overline{AR}_{nt} } \right)}}$$

where N is the number of events in the sample for each country. For example, if a country has announced three QE programmes over the sample period, then N will be 3. ${\overline{AR} }_{nt}$ is the average of the excess return over the event window, and it is calculated as follows:

8 $$\overline{AR}_{nt} = \frac{1}{{n_{t} }}\mathop \sum \limits_{h \in n} AR_{t,h}$$

where n denotes the number of observations in the event window and ${AR}_{t,h}$ is the average return on day h within the event window. The analysis also covers the impact of policy announcement on a single day on and around the announcement day. The day 0 test statistic is given by the following equation:

9 $$T_{P,0} = \frac{1}{\sqrt N }\mathop \sum \limits_{i = 1}^{i = N} \frac{{AR_{i0} }}{{PE\left( {AR_{0} } \right)}}$$

The t-test is used to reject null with a confidence level if the test statistic is greater than the critical value. The validity of the test depends critically on the assumption that returns are normally distributed (Corrado and Zivney 1992). This assumption is relaxed for the non-parametric tests. Non-parametric tests such as the rank and the sign tests perform better, and they are more powerful in multi-day event windows (Campbell et al. 2010).

b. **Rank Test**

The advantage of the non-parametric tests over parametric t-tests is that they can identify small levels of ARs, and they require robustness against non-normally distributed data (Corrado 2011). In the first step, the Corrado’s (1989) rank transforms ARs into ranks, and this ranking is done for all ARs in both the event and the estimation period. Let *K*_0_ denote the rank of the event date standardised AR SAR_0_, within a sample of *m* + *n*, where m is the number of days in the estimation period, and n is the number of days in the event window. The equation is represented as follows:

10 $$K_{0} = {\text{rank}}\left( {SAR_{0} } \right),$$

The ranks are later used to compute the Corrado–Zivney rank test statistic. When analysing a multi-day event period, Corrado and Truong (2008) defined the rank test considering the sum of the mean excess rank for the event window as follow:

11 $$T_{cz} = \frac{1}{\sqrt N }\mathop \sum \limits_{i = 1}^{N} \frac{{\mathop \sum \nolimits_{h \in n,i} K_{i,h} - n\left( {\frac{n + m + 1}{2}} \right)}}{{\sqrt {nm\left( {n + m + 1} \right)/12} }}$$

where K_i,h_ is the rank in the event day across all events, n + *m* is the number of non-missing variables in the estimation window and event window, and it is standardised by its mean represented as $n(\frac{n+m+1}{2})$ and variance as $nm(n+m+1)/12$ (Hettmansperger 1984; Corrado and Truong 2008). Alternatively, the rank test may take the following form for the cumulative abnormal returns (CARs);

12 $$T_{R} = \frac{1}{\sqrt N }\mathop \sum \limits_{i = 1}^{N} \frac{{\overline{K}_{i,t} - n\left( {\frac{n + m + 1}{2}} \right)}}{{\sqrt {nm\left( {n + m + 1} \right)/12} }}$$

where ${\overline{K} }_{i,t}$ is denotes the sum of standardized excess returns in the event window (by using Eq. 6). The letter *n* is the number of days in the event window starting from the fifth day before the event day and ending on the fifth day after the event day, a total of 11 days for the event window. Additionally, 7- and 3-day event windows are also employed in the analysis.

The day 0 test statistic is given by Corrado and Zivney (1992) as follows:

13 $$T_{R,0} = \frac{1}{\sqrt N }\mathop \sum \limits_{i = 1}^{N} \frac{{K_{i,0} - \left( {\frac{m + 1}{2}} \right)}}{{\sqrt {m\left( {m + 1} \right)/12} }}$$

The difference between Eqs. 12 and 13 (as well as Eqs. 7, 9) is that Eq. 12 (Eq. 7) provides test statistics for CARs with different event windows, where Eq. 13 (Eq. 9) is used to calculate t-test for each individual day in the event window. This helps to identify ARs for exchange rates either daily or cumulatively over a multiday event window. CAR is useful in event studies when the event day is not exactly known, as returns are cumulated over an interval that encompasses the actual event (Kolari and Pynnonen 2011). The CB announcements for policy changes are based on the press releases. The information may be shared in the market prior to the official release, usually a few days before the official date, hence making cumulative analysis more important.

c. **Sign Test**

The sign test is commonly used to specify statistical significance independently of an assumption concerning the distribution of the excess return population from which data are collected (Corrado and Zivney 1992). The null hypothesis that the shift in the distribution of event date excess returns is zero. Corrado and Truong (2008) propose two generalisations of the sign test to allow for non-symmetric excess-return distributions as follows:

14 $$T_{S} = \frac{1}{\sqrt N }\mathop \sum \limits_{i = 1}^{N} \frac{{\left( {\mathop \sum \nolimits_{h \in n} G_{i,h} - n\frac{1}{m}\mathop \sum \nolimits_{h \in m} G_{i,h} } \right)}}{{\sqrt {n\frac{1}{m}\mathop \sum \nolimits_{h \in m} G_{i,h} \left( {1 - \frac{1}{m}\mathop \sum \nolimits_{h \in m} G_{i,h} } \right)} }}$$

where *G* represents the sign of the AR. When *AR* > 0, meaning that actual rate is greater than the predicted return, resulting in a depreciation of the domestic currency. When *AR* has a negative sign, it means that the domestic currency has moved away from its fundamental value, resulting in an appreciation of the domestic currency. The empirical evidence provides mixed results about the impact of QE announcements on domestic exchange rate, and the results are highly dependent on the credibility and the effectiveness of the monetary policy strategy (Kenourgios et al. 2015). This study will be using the sign test to determine whether QE announcements cause domestic appreciation. In this respect, *G* will take the value of 1 if *AR* < 0, and 0 otherwise.^4^ Finally, the equation takes the following form for the day 0 test statistics:

15 $$T_{S,0} = \frac{1}{\sqrt N }\mathop \sum \limits_{i = 0}^{N} \frac{{\left( {G_{i,0} - \frac{1}{m}\mathop \sum \nolimits_{h \in m} G_{i,h} } \right)}}{{\sqrt {\frac{1}{m}\mathop \sum \nolimits_{h \in m} G_{i,h} \left( {1 - \frac{1}{m}\mathop \sum \nolimits_{h \in m} G_{i,h} } \right)} }}$$

Equation 14 tests cumulative ARs whereas Eq. 15 tests ARs for each day within the event window to keep the consistency with the previous tests. The estimation results of the parametric t-test, the rank test and the sign test are all explained in the following section.

d. **Estimation Results**

The next step is to assess the ability of the three test statistics to detect any abnormal performances after announcements during the event window. Tables 5 and 6 show the parametric and non-parametric test results to detect ARs for the US dollar and euro as the foreign currency. The impact of the QE on asset markets varies across emerging economies (Kočenda and Moravcová 2018; Pandey and Kumari 2021). The results for the cumulative tests provide statistically significant results in all countries, which is consistent with the literature (see Boubaker et al. 2022; Pandey and Kumar 2022; Pandey and Kumari 2021). In addition to this, the magnitude of the t-test results show that the impact is stronger for larger event windows (Krishnamurthy and Vissing-Jorgensen 2011; Hayword 2018; Pandey and Kumari 2021). The largest AR can be seen in Hungary for the USD for 11-day event window. In fact, larger magnitudes for ARs in Hungary are consistent with other empirical findings too. This study finds that after domestic QE Hungarian Forint provided larger AR than Polish Zloty. Similarly, Kočenda and Moravcová (2018) use the rank test to measure the impact of the US and the ECB QE on short term changes of exchange rates in Hungary and Poland. They also find that Hungarian Forint provided larger ARs than Polish Zloty around the announcement of QE by the ECB.

**Table 5:** The effect of QE announcement on AR for the USD exchange rate

	Hungary				Poland				Turkey			
	AR (%)	T_P_	T_R_	T_S_	AR (%)	T_P_	T_R_	T_S_	AR (%)	T_P_	T_R_	T_S_
t = − 5	0.0031	2.2206**	1.2458	− 1.7484*	0.0026	− 0.1947	− 0.4984	1.5119	− 0.0019	0.9208	− 0.6156	0.8730
t = − 4	0.0103	4.1680***	2.0703**	− 1.7484*	0.0001	− 0.4835	0.1824	1.8898*	− 0.0041	1.4412	− 1.1619	0.8730
t = − 3	0.0027	1.8755**	0.0836	− 0.5937	− 0.0033	− 0.8764	− 2.7622***	1.8898*	0.0073	− 0.6939	1.6488*	− 1.1455
t = − 2	0.0114	5.8022***	1.5337	− 1.7484*	− 0.0019	0.2571	0.3062	1.5119	− 0.0031	1.4412	− 0.9451	0.8730
t = − 1	− 0.0072	− 2.2735***	− 2.3308**	0.5611**	− 0.0071	− 1.9753**	− 3.0652***	2.6458***	0.0046	− 0.6939	1.5261	− 1.1455
t = 0	− 0.0038	− 2.0789**	− 0.3712	0.5611	− 0.0009	− 0.2486	− 0.5896	1.8898*	0.0056	− 0.6939	1.5695	− 1.1455
t = 1	0.0016	0.8374	0.0721	0.5611	0.0010	− 0.4195	− 0.7785	1.5119	0.0007	1.4412	0.5550	− 1.1455
t = 2	− 0.0053	− 1.6719*	− 2.0273**	1.7158*	0.0023	0.9136	2.2411**	0.3780	− 0.0027	1.4412	− 0.8411	0.8730
t = 3	− 0.0033	− 0.4657	− 0.2042	− 0.5937	− 0.0009	− 0.3032	− 0.5212	1.5119	0.0032	− 0.6939	1.3007	− 1.1455
t = 4	0.0108	− 0.3307	1.8510*	− 0.5937	0.0006	− 0.5823	− 0.9121	2.2678**	0.0018	1.4412	0.9451	− 1.1455
t = 5	0.0065	− 0.5053	1.6433	− 0.5937	− 0.0031	0.3072	0.4788	1.1339	− 0.0008	− 0.6939	− 0.1561	0.8730
CAR 3	− 0.0094	− 6.7423***	1.0717	− 3.7098***	− 0.0069	− 2.6376***	1.4442	− 1.8911*	0.0109	0.7263	2.0837**	− 1.9840**
CAR 7	− 0.0039	2.8082***	5.4827***	− 1.9690**	− 0.0107	− 2.7064***	7.1951***	− 1.5513	0.0155	1.0302	4.4769***	− 1.5048
CAR 11	0.0268	12.0266***	11.1356***	− 1.2726	− 0.0105	− 3.6505***	12.3178***	− 2.6306***	0.0105	0.7676	6.2959***	− 0.7562

T_S_ is T-test, T_R_ is Rank test and T_S_ is Sign test

AR refers to abnormal returns

If the day of the policy announcement is represented by t = 0, then t − 1 will be one day before the announcement and t + 1 will be one day after the announcement

*,**,***Denote statistically significance at 10%, 5% and 1%, respectively

**Table 6:** The effect of QE announcement on AR for euro exchange rate

	Hungary				Poland				Turkey			
	AR (%)	T_P_	T_R_	T_S_	AR (%)	T_P_	T_R_	T_S_	AR (%)	T_P_	T_R_	T_S_
t = − 5	0.0003	1.7587*	0.5970	− 1.8096**	0.0017	− 0.1209	− 0.6971	1.8898*	− 0.0016	0.3922	− 0.5346	0.9208
t = − 4	0.0080	4.9506***	2.0179**	− 1.8096**	− 0.0001	− 0.1346	− 0.1954	1.5119	− 0.0010	0.1561	− 0.2999	1.4412
t = − 3	0.0012	1.7318*	0.5664	− 0.6538	− 0.0003	− 0.9152	− 1.8176*	1.8898*	0.0075	0.2658	1.6473*	− 0.6939
t = − 2	0.0065	6.5432***	1.5976	− 1.8096**	− 0.0003	0.1711	0.6254	0.7559	− 0.0025	− 0.1733	− 0.8823	1.4412
t = − 1	− 0.0084	− 4.8117***	− 2.8029***	1.6579*	− 0.0007	− 0.9783	− 1.4072	1.5119	0.0037	0.2579	1.4039	− 0.6939
t = 0	0.0015	− 0.1079	− 0.1414	0.5021	0.0007	0.7966	1.3779	1.1339	0.0036	0.2491	1.3865	− 0.6939
t = 1	0.0023	2.3009**	− 0.8316	− 0.6538	0.0004	− 0.3783	− 0.4137	1.1339	− 0.0012	− 0.0800	− 0.3347	1.4412
t = 2	− 0.0022	− 0.7078	− 1.2746	0.5021	− 0.0002	0.4204	0.8599	1.1339	− 0.0011	0.1278	0.9171	1.4412
t = 3	− 0.0036	− 3.1016***	− 1.9360*	1.6579*	− 0.0003	− 0.0752	0.2997	0.7559	0.0018	0.2000	1.2387	− 0.6939
t = 4	0.0030	− 2.5882***	2.4487**	− 0.6538	0.0002	− 0.4743	− 1.1401	1.8898*	− 0.0017	0.1109	− 0.6128	1.4412
t = 5	0.0038	− 1.8017*	1.5915	− 0.6538	0.0001	0.2054	− 0.0195	1.5119	0.0029	− 0.1448	1.2387	− 0.6939
CAR 3	− 0.0046	− 2.8127***	1.4738	− 1.2928	0.0003	− 0.5591	2.6454***	0.2265	0.0060	0.4210	1.7182**	− 0.7223
CAR 7	− 0.0027	2.7420***	6.1150***	0.4546	− 0.0008	− 0.9511	8.2255***	0.1555	0.0117	0.8116	4.1755***	− 0.5977
CAR 11	0.0123	11.2514***	10.8034***	− 1.1228	0.0011	− 1.4651	12.8619***	− 0.3261	0.0103	0.7068	6.2003***	0.0287

T_S_ is T-test, T_R_ is Rank test and T_S_ is Sign test

AR refers to abnormal returns

If the day of the policy announcement is represented by t = 0, then t-1 will be one day before the announcement and t + 1 will be one day after the announcement

*^,^**^,^***Denote statistically significance at 10%, 5% and 1%, respectively

Furthermore, the ARs on a daily analysis provide statistically significant results during the pre-announcement period when it is compared to the post announcement period. This is also consistent with other studies such as Fatum and Hutchison (2003) and Kočenda and Moravcová (2018), which argue that the markets react not only after the news release, but they react before as well. This can be explained by the fact that the analysis assumes the event day (t = 0) on the day of press. In most cases, the markets may have access to information a few days before it is officially published. In fact, the results show that the QE announcements do not provide ARs on the day of the announcement on many occasions.

The response of the exchange rate to the policy announcement is sensitive to the tests used to assess the average AR on an event period and accumulated AR around the event period (Kothari and Warner 2006). Hungary performs better in providing statistically significant ARs for both parametric and non-parametric t-tests for both currencies, while Poland has better test specification for both parametric and non-parametric t-tests only for the USD. For euro, the non-parametric tests have better test specification. On the other hand, only the rank tests are meaningful for CAR analysis in Turkey, and the reaction is very similar for both currencies.

CARs perform better with respect to limiting Type II error (accepting a null hypothesis when it is false). The Patell t-test provide statistically significant ARs for the USD exchange rate in Hungary and Poland. Furthermore, AR for euro exchange rate is statistically significant only in Hungary. The ranks tests in all countries provide statistically significant ARs for euro exchange rate and for all estimation windows in Poland and Turkey. It provided statistically significant ARs for all windows in Turkey and only for 7- and 11-day window in Hungary. The impact of the news is stronger for the US dollar in Hungary and for euro in Poland, which is consistent with the study of Kočenda and Moravcová (2018).

The sign of the ARs represented in the first column for each country in Tables 5 and 6 are consistent with the results of the sign test, with the only exception being Turkey for the USD for a 3-day window. The sign test provides statistically significant ARs only for the USD exchange rate in all countries, and ARs are mostly statistically significant in shorter event windows. In other words, QE announcements cause domestic appreciation against the USD. QE causes appreciation of the domestic currency against the USD for a 3-day window in all countries. Sign tests are not statistically significant for euro in any of the three countries in any of the event windows.

Finally, for robustness the response of local-currency government bond yields to QE announcements are provided in “Appendix A.3”. The results show that announcements reduce the bond yields in all countries for all windows, which is consistent with theory and empirical results (Palley 2011; Gagnon et al. 2011; Krishnamurthy and Vissing-Jorgensen 2011; Hartley and Rebucci 2020). The test results are stronger in Hungary and Turkey for a 3-day window, and it is strongest in Poland for a 7-day window. Parametric tests perform better in short event windows and non-parametric tests perform better in longer event windows.

## Measuring event-induced volatility: the exponential GARCH model

One of the major characteristics of the financial time series is that they have intervals of large volatility that makes the assumption of stable variance invalid. Engle (1982) introduced the Autoregressive Conditional Heteroscedasticity (ARCH) model for the financial time series that exhibit time varying conditional variance. The Generalised Autoregressive Conditional Heteroskedastic (GARCH) model introduced by Bollerslev (1986) extended Engle’s original work of ARCH process to allow conditional variance to be an ARMA process.

The general form of the GARCH (p,q) model is given as follows:

16 $$er_{t} = \mu + \varepsilon_{t}$$

17 $$log\sigma_{t}^{2} = \omega + \mathop \sum \limits_{i = 1}^{q} \alpha \varepsilon_{t - i}^{2} + \mathop \sum \limits_{j = 1}^{p} \beta_{j} log\sigma_{t - j}^{2}$$

Equation 16 represents the mean equation, where *er* is the exchange rate, *μ* is the expected exchange rate, and *ε* is the error term. Equation 17 represents the volatility equation including the lagged terms of the squared error terms, *ε*^2^, and lagged conditional variance, σ^2^. The model has the assumptions that parameters *ω, α*_*i*_*,* and *β*_*j*_ are to be positive, and the parameter *ω* is expected to be small. Parameter *α*_*i*_ is part of the ARCH component measuring the response of volatility on market variances, and parameter *β*_*j*_ is part of the GARCH component showing the difference caused from outliers on conditional variance. Finally, the model expects the sum *α*_*i*_ + *β*_*j*_, which shows the impact of the variables’ variance of the previous period regarding the current value of volatility, to be less than one. A value very close to one is a sign of increasing inactivity of shocks of the volatility of returns on the financial assets (Dritsaki 2017).

Exchange rates tend to react differently to policy changes either through conventional policies such as interest rates (Salisu et al. 2021) or unconventional policies such as foreign exchange interventions (Lahura and Vega 2013). Thus, asymmetric behaviour of exchange rates, becomes important for the model that will be assessed in this study. One of the most popular asymmetric ARCH models is the Exponential GARCH (EGARCH) model proposed by Nelson (1991). The EGARCH model has two key advantages over GARCH model. Firstly, the model uses log returns, therefore, even if the parameters are negative the conditional variance will be positive. Secondly, the logarithmic expression of the conditional volatility will be used to capture the asymmetric effects (Pilbeam and Langeland 2015).

18 $$log\sigma_{t}^{2} = \omega + \mathop \sum \limits_{i = 1}^{q} \alpha_{i} \left| {\frac{{\varepsilon_{t - i} }}{{\sigma_{t - i} }}} \right| + \mathop \sum \limits_{j = 1}^{p} \beta_{j} log\sigma_{t - j}^{2} + \mathop \sum \limits_{k = 1}^{r} \gamma_{k} \frac{{\varepsilon_{t - k} }}{{\sigma_{t - k} }}$$

*ω, α*_*i*_*, β*_*j*_ and *γ*_*k*_ are parameters that can be estimated using the maximum likelihood method. Parameter *ω* represents the long-term average value. Parameter *α*_*i*_ measures the response of volatility on market variance and parameter *β*_*j*_ measures the difference, which is caused from outliers on conditional variance (Dritsaki 2017). Additionally, the model assumes *α*_*i*_ < 1 and *β*_*j*_ < 1 as a requirement for achieving stationarity on the variance. The EGARCH model uses the level of standardised value of ${\varepsilon }_{t-k}$, and parameter *γ*_*k*_, which is also called the leverage effect, and it is mainly added to the equation to capture the asymmetric shocks. Nelson (1991) argues that this standardisation allows for a more natural interpretation of the size and persistence of shocks. It is considered that *ε*_*t-k*_ term is the one that establishes the asymmetry of EGARCH *(p,q)* when parameter γ_*k*_ ≠ 0. A general explanation is that past negative shocks have a deeper impact on current conditional volatility than past positive shocks (see Black 1976; Christie 1982). This also means that positive shocks cause short term volatility and instability in relation to negative shocks (Dritsaki 2017). Henceforth, we expect volatility to increase after a negative shock and decrease after a positive shock. Since volatility tends to rise in response to bad news and fall in response to good news, the exchange rates may display higher volatility during periods of depreciation compared to periods of appreciation. The exchange rates are represented as domestic currency per foreign currency, and reductions are associated with appreciations whereas increases are associated with depreciation. The relationship between foreign exchange returns and volatility can be expressed as follows: If the relationship between volatility and returns are positive, *γ* will be positive, thus implying bad news (depreciation) generates more volatility than good news (appreciation). If the relationship is negative, then *γ* will be negative, implying that good news (appreciation) generates more volatility than bad news (depreciation). Finally, any shock will lead to a permanent change in all future values if *α*_*i*_ + *β*_*j*_ = 1. Hence, the shock of conditional variance is persistence.

The most appropriate model in the volatility estimation for the EGARCH (1,1) model is represented in the following form:

19 $$log\sigma_{t}^{2} = \omega + \alpha_{1} \left| {\frac{{\varepsilon_{t - 1} }}{{\sigma_{t - 1} }}} \right| + \beta_{1} log\sigma_{t - 1}^{2} + \gamma_{1} \frac{{\varepsilon_{t - 1} }}{{\sigma_{t - 1} }}$$

The model uses the level of standardised value of ${\varepsilon }_{t-1}$, i.e., ${\varepsilon }_{t-1}$ divided by ${\sigma }_{t-1}$. Nelson (1991) argues that this standardising allows for a more natural interpretation of the size and persistence of shocks. The objective is to investigate the volatility pattern of emerging Europe exchange rates and the impact of QE announcement on volatility.

Table 7 shows the summary statistics for the exchange rates at a daily frequency. It shows that the level of kurtosis is higher in all dollar exchange rates than euro for Hungary and Poland. The level is highest in Poland, indicating that extreme changes tend to occur more frequently for dollar than euro. Skewness is present in all countries for both currencies, and positive skew shows the fatter tail on the right side of the distribution. The Jarque–Bera (JB) statistics rejects normality at the 1% level for all currencies, unit root and stationary tests (not presented here) show that all exchange rates are nonstationary, (1). Thus, the estimations include exchange rates in natural logarithm and in the first difference.

**Table 7:** Descriptive statistics

		Mean	Median	Maximum	Minimum	SD	Skewness	Kurtosis	JB
Hungary	USD	295.28	295.25	337.46	270.01	13.26	0.52	2.87	35.07
	Euro	338.11	332.77	369.03	312.74	16.27	0.34	1.56	81.58
Poland	USD	3.83	3.80	4.27	3.62	0.13	1.32	4.97	350.11
	Euro	4.38	4.32	4.66	4.23	0.11	0.59	1.88	84.80
Turkey	USD	6.36	6.01	8.55	4.47	1.00	0.42	2.14	47.41
	Euro	7.31	6.69	10.42	5.27	1.32	0.68	2.16	82.52

Tables 8 and 9 present the estimation results for EGARCH (1,1) specification for both the US dollar and the euro. The assumptions of the model, where *α*_*i*_ < 1 and *β*_*j*_ < 1, are held in all estimates. Parameters *α* and *β* are statistically significant in all countries for euro and all countries except Hungary with a 3-day window. In all countries for euro as the foreign currency, the sum of *α* and *β* is either very close to 1 or greater than 1. For dollar exchange rate, the sum is greater than 1 in Poland and Turkey. This means that any shock will lead to a permanent change in all future values, except dollar exchange rate in Hungary. One of the important parts of the EGARCH method is to examine the residuals for the evidence of heteroskedasticity. Lagrange multiplier (LM) test for ARCH has a null hypothesis that there is no ARCH. The results show that the null is rejected in all countries for all currencies ensuring the estimations are correctly estimated, except Turkey (for USD with 7-day window). Furthermore, t-student degrees of freedom parameter are all statistically significant.

**Table 8:** Parameter estimates for the USD

	Hungary			Poland			Turkey		
	3 Days	7 Days	11 Days	3 Days	7 Days	11 Days	3 Days	7 Days	11 Days
*Mean equation*									
μ	0.0000	0.0000	0.0000	0.0000	0.0000	0.0002	0.0002	0.0000	0.0002
	(0.000)	(0.000)	(0.000)	(0.000)	(0.000)	(0.000)	(0.000)	(0.000)	(0.000)
QE dummy	− 0.0006	− 0.0019	0.0005	− 0.0022*	− 0.0015*	− 0.0012*	0.0053**	0.0044***	0.0021
	(0.002)	(0.002)	(0.002)	(0.001)	(0.001)	(0.001)	(0.002)	(0.001)	(0.002)
*Variance equation*									
ω	− 8.4816***	− 3.8225**	− 6.0155***	− 0.2414*	− 0.1837***	− 0.6150**	− 0.7738***	− 2.6794***	− 0.7859***
	(2.677)	(1.907)	(2.346)	(0.134)	(0.049)	(0.297)	(0.162)	(0.073)	(0.163)
α	0.0560	0.1263**	0.0369	0.0912***	0.0865***	0.1194***	0.3828***	0.1911***	0.5795***
	(0.089)	(0.071)	(0.084)	(0.026)	(0.024)	(0.071)	(0.039)	(0.036)	(0.084)
β	0.1749	0.6296***	0.4189*	0.9835***	0.9892***	0.9495***	0.7573***	0.7288***	0.9566***
	(0.262)	(0.189)	(0.227)	(0.009)	(0.004)	(0.028)	(0.015)	(0.080)	(0.059)
γ	0.7602**	0.0937	0.0828	0.0142	0.0281	0.0156	− 0.1847***	− 0.0880***	− 0.0039
	(0.516)	(0.046)	(0.057)	(0.021)	(0.018)	(0.025)	(0.006)	(0.025)	(0.045)
QE Dummy	0.7618	0.3355	0.6453**	0.0338	0.0290	0.0425	− 0.8316***	− 2.7944***	− 0.1879
	(0.488)	(0.257)	(0.288)	(0.070)	(0.023)	(0.032)	(0.225)	(0.313)	(0.278)
df	8.0324***	8.6115***	9.9695***	11.5405***	10.7317***	10.6720***	6.2111***	4.8041***	3.2587***
	(2.116)	(2.372)	(3.289)	(3.561)	(3.077)	(2.138)	(0.707)	(0.504)	(0.437)
AIC	− 7.384	− 7.396	− 7.353	− 7.421	− 7.369	− 7.371	− 6.610	− 6.472	− 6.763
SIC	− 7.336	− 7.348	− 7.383	− 7.374	− 7.398	− 7.400	− 6.562	− 6.419	− 6.715
Observations	781	781	781	781	781	781	781	781	781
Backcast with parameter	0.5	0.5	0.6	0.5	0.4	0.5	0.5	0.4	0.4
α + β	0.23	0.76	0.46	1.07	1.08	1.07	1.14	0.92	1.54
α + **γ**	0.82	0.22	0.12	0.11	0.11	0.13	0.20	0.10	0.58
ARCH (1)	0.15	0.17	0.33	0.37	0.56	0.01	0.00	11.43***	0.00

Optimal lags of the model are selected by Akaike (AIC) and Schwarz (SIC) information criteria

Event windows: 3 days refer to ± 1 day, 7 days refer to ± 3 days and 11 days refer to ± 5 days around the announcement day

df refers to t-student degrees of freedom parameter

The ARCH test provides F-stat results for heteroskedasticity test, and the number of lags is given in parenthesis

Backcasting presents the smoothing parameter used in variance estimation. When the parameter is set to 1, it presents unconditional variance So that backcasting is not calculated

*^,^**^,^***Denote statistically significance at 10%, 5% and 1%, respectively

**Table 9:** Parameter estimates for the euro

	Hungary			Poland			Turkey		
	3 Days	7 Days	11 Days	3 Days	7 Days	11 Days	3 Days	7 Days	11 Days
*Mean equation*									
μ	0.0000	0.0001	0.0000	0.0000	0.0000	0.0000	0.0007***	0.0007***	0.0007***
	(0.000)	(0.000)	(0.000)	(0.000)	(0.000)	(0.000)	(0.000)	(0.000)	(0.000)
QE dummy	− 0.0001	− 0.0012	0.0001	− 0.0001	− 0.0004	− 0.0003*	0.0013	0.0029	0.0017
	(0.001)	(0.001)	(0.001)	(0.001)	(0.000)	(0.000)	(0.002)	(0.000)	(0.002)
*Variance equation*									
ω	− 0.8484***	− 1.8317***	− 4.1749***	− 4.5672***	− 2.2219***	− 1.5984***	− 0.9496***	− 0.9913***	− 0.9710***
	(0.019)	(0.536)	(1.217)	(0.109)	(0.050)	(0.146)	(0.227)	(0.236)	(0.232)
α	0.2881***	0.2642***	0.2625***	0.3081***	0.3312***	0.3739***	0.4379***	0.4486***	0.4445***
	(0.046)	(0.065)	(0.083)	(0.053)	(0.040)	(0.059)	(0.070)	(0.072)	(0.071)
β	0.9459***	0.8570***	0.6508***	0.6374***	0.8367***	0.8893***	0.9303***	0.9265***	0.9283***
	(0.002)	(0.046)	(0.106)	(0.007)	(0.003)	(0.010)	(0.022)	(0.023)	(0.022)
γ	0.0906***	0.0849**	0.1004**	− 0.0455	0.0106	0.0805**	0.0206	0.0188	0.0175
	(0.029)	(0.033)	(0.049)	(0.037)	(0.031)	(0.019)	(0.039)	(0.040)	(0.040)
QE dummy	− 0.1452	0.1803	0.4073**	0.4371**	0.1605**	0.0952*	− 0.2803	− 0.1527	− 0.0895
	(0.158)	(0.139)	(0.206)	(0.241)	(0.082)	(0.059)	(0.549)	(0.202)	(0.135)
df	14.4051**	14.8887***	12.5289**	14.7829***	13.4004***	6.5194***	3.6399***	3.6172***	3.6157***
	(6.811)	(6.752)	(5.585)	(3.979)	(4.664)	(1.643)	(0.501)	(0.498)	(0.497)
AIC	− 8.467	− 8.463	− 8.442	− 8.885	− 8.939	− 8.966	− 6.672	− 6.675	− 6.673
SIC	− 8.419	− 8.415	− 8.394	− 8.837	− 8.891	− 8.918	− 6.625	− 6.627	− 6.626
Observations	781	781	781	781	781	781	781	781	781
Backcasting	0.5	0.5	0.5	0.5	0.5	0.2	0.5	0.5	0.5
α + β	1.23	1.12	0.91	0.95	1.17	1.26	1.37	1.38	1.37
α + **γ**	0.38	0.35	0.36	0.26	0.34	0.45	0.46	0.47	0.46
ARCH(1)	0.86	0.76	1.15	0.96	0.11	0.02	0.06	0.04	0.05

Optimal lags of the model are selected by Akaike (AIC) and Schwarz (SIC) information criteria

Event windows: 3 days refer to ± 1 day, 7 days refer to ± 3 days and 11 days refer to ± 5 days around the announcement day

df refers to t-student degrees of freedom parameter

The ARCH test provides F-stat results for heteroskedasticity test and the number of lags is given in paranthesis

Backcasting presents the smoothing parameter used in variance estimation

Backcasting presents the smoothing parameter used in variance estimation. When the parameter is set to 1, it presents unconditional variance so that backcasting is not calculated

*^,^**^,^*** denote statistically significance at 10%, 5% and 1%, respectively

Parameter *γ* represents the leverage effect required to capture asymmetry if the coefficient is statistically significant. The results show that asymmetry prevails in all countries, but this asymmetry is sensitive to the currency and the size of the window. For example, in Hungary the leverage effect is found statistically significant in euro estimates for all time intervals, and it is significant for only a 3-day window in dollar estimate. The positive sign shows that depreciation of the domestic currency causes more volatility than appreciation of the domestic currency. In Poland, the leverage effect is significant for euro for an 11-day window with a positive sign. Dollar estimates in Turkey shows that *γ* is statistically significant both for 3-day and 7-day windows, and negative values show that positive shocks (domestic appreciation) cause more volatility in relation to negative shocks. One explanation is that the Turkish lira has been exposed to continuous depreciation. Since 2017, due to political turmoil and the conflict with the US, Turkey has gone through a period of increased volatility and economic uncertainty. In the first half of 2018, global investors backed away from all emerging markets, and from June 2018 to May 2021, the Turkish lira lost approximately 82% of its value against the US dollar. Hence, during the period of constant depreciation, any appreciation might have triggered depreciation expectations subsequently causing more volatility in the foreign exchange markets.

The focus of the EGARCH method used in the analysis is to measure the impact of QE announcements on exchange rates and to investigate whether the policy announcements cause any changes in the volatility of the exchange rates in emerging European countries. The estimates include QE dummy in mean equation to measure the impact of QE policy announcements on exchange rates and a variance equation to measure its impact on volatility. The results for the dollar estimates show that the dummy coefficients of the mean equation are statistically significant for all time intervals in Poland, whereas in Turkey they are only statistically significant for 3-day and 7-day windows. However, for the euro estimates, the dummy coefficient is statistically significant only in Poland for an 11-day window. The sign of the coefficients is consistent with literature, providing mixed results for different countries and for different announcements (see for example,Neely 2011; Kenourgios et al. 2015; Fratzscher et al. 2013). The negative sign in Poland shows appreciation of the domestic currency against the US dollar and euro. These results are consistent with the results of the event study in Sect. 4. The positive sign for Turkey shows depreciation of the domestic currency against the US dollar around the days of QE policy announcement. These signs are persistent for longer time intervals. Furthermore, for an 11-day window the dummy coefficient in the variance equation is statistically significant in Hungary for both currencies, and it is statistically significant in Poland for all time intervals but just for euro. The positive sign indicates an increased volatility around the QE announcement. In Hungary, announcements increase volatility for the US dollar in an 11-day window. In Poland, announcements increase volatility for euro in all estimation window. However, in Turkey QE announcement reduces volatility for euro in 3- and 7-day windows.

## Conclusion

There is no doubt that the recent pandemic has affected our daily lives and the well-being of the global economy. However, the impacts on financial markets, has been less straightforward. The response of financial markets depends highly on the size of fiscal and monetary stimulus packages provided by authorities around the world. The impact of the pandemic on emerging economies were more severe due to their economic and political instabilities and their limited financial resources. The response of CBs in emerging markets was geared towards removing instability during the pandemic. In order to secure investors’ confidence and to maintain stability in the financial markets CBs in EEE used quantitative easing together with other conventional and unconventional methods.

Since the event studies are useful in measuring the investors’ confidence, the methodology used in this study shows that QE announcements affects the exchange rates in the short run. The QE announcements are causing ARs in foreign exchange rates resulting in an appreciation of the domestic currency in Hungary and Poland and a further depreciation in Turkey. In terms of securing investors’ confidence, the study shows that the new policy instrument is successful in Hungary and Poland, but not necessarily in Turkey. Finally, the volatility tests are useful in measuring stability in foreign exchange markets. The results also confirm that QE announcements during the pandemic increased volatility in Hungary and Poland, but reduced volatility in Turkey. Increased volatility has many implications on trade flows, firms’ operating cash flows, and cost and market structure variables. The results for the exchange rate volatility show that asymmetry prevails in all countries, but this asymmetry is sensitive to the currency and the length of the window.

The main result from the analysis is that unconventional policy in emerging European markets in response to an economic shock has significant effect on financial variables beyond Treasury yields. This result has important implications that are relevant for policymakers for understanding the changes in market expectations after a change future monetary policy stance. Under the conditions of stress, the effects of QE policies on financial markets are amendable to direct observation through event studies. It is important for central banks to be informed about the way their communication channel works during a crisis, and how their policy announcements affect expectations in foreign exchange markets. However, further analysis is required to observe the effects and the transmission channels of QE beyond the short-term, and to assess whether QE is an effective policy instrument.

## Appendix A

### Appendix A.1: Data

Set of interventions includes 25 QE announcements of the CBs in five EEE; Croatia, Hungary, Poland, Romania, and Turkey. The time span covered in the analysis is from 1 June 2018 to 31 May 2020, including the COVID-19 Pandemic starting officially on 30 May 2020, at a daily frequency. The QE announcements for each national CB were obtained from the press releases after the Monetary Council meetings. The analysis includes the impact of these press releases on domestic currency against the US dollar and euro. The analysis also includes the impact of QE announcements on 10-year government bond yields for checking the robustness of the results for the event study.

All data except interest rates are in log-difference stationary series of percentage exchange rate returns and calculated as *er*_*t*_ = log(*e*_*t*_/*e*_*t–1*_), where *er* is the logarithmic exchange rate series in first difference. The exchange rates and interest rates are obtained from CBs. Increases are associated with depreciation of domestic currency. 10-year government bond yields are obtained from Investing.com.

### Appendix A.2 : Cumulative percentage change in 10-year government bond yield

See Table 10.

**Table 10:** Cumulative percentage change in 10-year government bond yield

Country		Date	11-Day window		7-Day window		3-Day window	
			Impact	SE	Impact	SE	Impact	SE
Croatia	1	13.03.2020	0.056***	0.009	0.088***	0.012	0.114***	0.018
Hungary	1	24.03.2020	− 0.021**	0.010	− 0.031***	0.012	− 0.083***	0.018
	2	28.04.2020	− 0.014	0.009	− 0.032**	0.013	− 0.065***	0.018
	3	21.07.2020	− 0.006	0.009	− 0.010	0.012	− 0.029*	0.018
	4	27.04.2021	0.007	0.010	0.002	0.012	0.001	0.018
Poland	1	17.03.2020	0.020**	0.008	0.008	0.010	− 0.066***	0.017
	2	08.04.2020	− 0.012	0.008	− 0.040***	0.014	− 0.040***	0.012
	3	28.05.2020	− 0.003	0.008	− 0.027**	0.011	− 0.060***	0.014
	4	16.06.2020	0.004	0.008	− 0.005	0.009	0.006	0.012
	5	14.07.2020	− 0.010	0.008	− 0.012	0.010	− 0.015	0.017
	6	15.09.2020	− 0.004	0.008	− 0.004	0.010	0.003	0.017
	7	07.10.2020	− 0.010	0.012	− 0.007	0.011	− 0.009	0.014
	8	06.11.2020	− 0.002	0.008	− 0.001	0.010	0.006	0.017
	9	02.12.2020	0.016**	0.008	0.029***	0.011	0.015	0.014
	10	13.01.2021	− 0.003	0.008	− 0.009	0.011	− 0.008	0.014
	11	03.02.2021	0.007	0.007	0.015*	0.009	0.022	0.014
	12	03.03.2021	0.016**	0.007	0.021**	0.009	0.013	0.014
	13	07.04.2021	0.000	0.008	− 0.002	0.010	− 0.001	0.017
	14	05.05.2021	0.019***	0.007	0.020**	0.009	0.001	0.014
Romania	1	20.03.2020	0.043***	0.007	0.081***	0.010	0.047	0.021**
	2	29.05.2020	− 0.009	0.007	− 0.013*	0.008	− 0.020	0.012*
	3	05.08.2020	− 0.005	0.007	− 0.010	0.008	− 0.024	0.012**
Turkey	1	31.03.2020	− 0.006	0.006	0.012	0.008	0.009	0.012
	2	17.04.2020	− 0.003	0.006	0.002	0.008	0.0105	0.0117

*^,^**^,^***Denote statistically significance at 10%, 5% and 1%, respectively

### Appendix A.3: The effect of QE announcement on AR for 10-year bonds

See Table 11.

**Table 11:** The effect of QE announcement on AR for 10-year bonds

	Hungary				Poland				Turkey			
	AR (%)	T_P_	T_R_	T_S_	AR (%)	T_P_	T_R_	T_S_	AR (%)	T_P_	T_R_	T_S_
t = − 5	0.0230	1.4010	1.3572	− 0.5504	0.0032	0.8877	2.1699**	− 0.6609	− 0.0008	− 0.0788	− 0.6885	1.4412
t = − 4	− 0.0038	− 0.2304	− 0.3416	0.6044	0.0054	0.0495	1.0522	− 1.4210	0.0189	1.8653*	1.6994*	− 0.6939
t = − 3	0.0113	0.6893	1.1110	− 0.5504	− 0.0045	− 0.9127	0.2937	0.0993	0.0174	1.7144*	1.6732*	− 0.6939
t = − 2	0.0096	0.5859	1.1725	− 1.7053*	− 0.0027	− 0.0610	− 0.9503	0.8595	0.0037	0.3638	0.2789	− 0.6939
t = − 1	− 0.0216	− 1.3179	− 2.0096**	1.7592*	− 0.0011	0.3998	− 0.2316	− 0.6609	0.0160	1.5815	1.6497*	− 0.6939
t = 0	− 0.0186	− 1.1320	− 0.8094	0.6044	− 0.0015	− 0.2521	1.4764	0.0993	− 0.0070	− 0.6949	− 1.3508	1.4412
t = 1	− 0.0135	− 0.8060	0.8401	− 0.5504	0.0033	1.1363	1.3493	− 0.6609	− 0.0586	− 5.4536***	− 1.7255*	1.4412
t = 2	− 0.0118	− 0.7206	− 2.1204**	1.7592*	0.0020	0.6583	− 0.0452	0.8595	0.0158	1.5563	1.6509*	− 0.6939
t = 3	− 0.0095	− 0.6527	− 0.9017	− 0.5504	− 0.0074	− 1.0983	− 2.2190**	2.3798**	− 0.0176	− 1.7300*	− 1.6558*	1.4412
t = 4	− 0.0040	− 0.2468	0.9509	− 1.7053*	0.0036	− 1.4305	− 2.6291***	0.8595	0.0045	0.4430	0.4967	− 0.6939
t = 5	0.0227	− 0.0809	1.6454*	− 0.5684	− 0.0006	0.5899	0.4934	− 0.6609	0.0011	0.1050	− 0.3137	− 0.6939
CAR 3	− 0.0536	− 4.9683***	1.2325	− 1.8928**	0.0007	1.4890	2.8919***	0.6109	− 0.0496	− 7.3291***	0.5849	1.2635
CAR 7	− 0.0540	− 3.3435***	4.6839***	0.7261	− 0.0120	− 0.9538	7.1314***	− 4.0470***	− 0.0303	− 2.9575***	3.2115***	0.5851
CAR 11	− 0.0160	0.0249	9.4595***	1.6493	− 0.0004	− 0.3924	12.1989***	− 6.7758***	− 0.0066	− 0.6456	5.6389***	0.2736

T_S_ is T-test, T_R_ is Rank test and T_S_ is Sign test

AR refers to abnormal returns

If the day of the policy announcement is represented by t = 0, then t − 1 will be one day before the announcement and t + 1 will be one day after the announcement

*^,^**^,^***Denote statistically significance at 10%, 5% and 1%, respectively

## Footnotes

1 Large scale asset purchases and quantitative easing will be used interchangeably.

2 Serbia also conducted QE as policy instrument during the pandemic. Since the policy was not explicitly reported in the minutes of central bank meetings, Serbia is excluded from the analysis.

3 “Appendix A.2” provides cumulative percentage change in 10-year government bond yields for these countries. The results are statistically significant, especially for shorter event windows, and the signs are consistent with the literature and the expectations.

4 The analysis also includes to test the impact of QE announcement for 10-year bond yields for robustness and it is expected that the announcement will increase bond prices and reduce bond yields. Since the analysis includes bond yields, so negative values for AR is still considered as 1 and 0 otherwise.
